# *Drosophila melanogaster*: an old and future ally to radiobiology

**DOI:** 10.1093/jrr/rraf060

**Published:** 2025-11-14

**Authors:** Terrence M Trinca, Joaquín de Navascués

**Affiliations:** School of Biochemistry, Biomedical Science Building, University Walk, University of Bristol, Bristol BS8 1TD, United Kingdom; School of Life Sciences, University of Essex, Wivenhoe Park, Colchester CO4 3SQ, United Kingdom

**Keywords:** *Drosophila melanogaster*, radiobiology, radiogenomics, radiation response, chronic radiation toxicity

## Abstract

From simple viruses to complex multicellular animals, ionizing radiation can have deleterious effects on all organisms. For humans, exposure to radiation can come from a wide range of sources such as environmental contamination, occupational hazards, radiotherapy and space flight. In the next few decades, radiation toxicity will become an increasing healthcare concern as nuclear power usage, risk of nuclear war, space-based industry and cancer incidence are all projected to increase. While the biology of acute radiation sickness is relatively well understood, ionizing radiation can also cause severe chronic effects whose molecular and cellular basis remain largely a mystery. This is partly because complications that arise months or even years after exposure depend on tissue-level responses, and so there are aspects of late radiation toxicity that can only be investigated *in vivo*. We suggest that *Drosophila melanogaster* can contribute to understanding this phenomenon. To this date, *Drosophila* radiation research has been heterogenous in terms of dose, radiation type and developmental stage of exposure, but despite this a pattern of observations suggest that fruit flies experience both short- and long-term radiation injury. Moreover, the genetic underpinning of the *Drosophila* radiation response seems conserved with that of humans. We propose that *Drosophila* is well-suited to model radiation damage to tissues, highlighting the potential of the fly to inform clinical radiobiology research.

## INTRODUCTION

Humans encounter ionizing radiation (IR) from many settings. There have been multiple environmental exposure incidents involving nuclear power plant disasters (Fukushima, Windscale Piles, Chernobyl and Mayak/Kyshtym), nuclear weapon development and warfare (Hiroshima, Nagasaki, Bikini Atolls and Nevada desert), and assassinations [1–5]. And besides these isolated incidents of high-level exposure, radiologists, civil aviation flight crews, astronauts and local populations near sites of nuclear testing/detonations can be chronically exposed to low levels of IR, creating occupational hazards and making some areas unsafe for prolonged habitation [6–9]. Cancer radiotherapy (RT) is by far the most common exposure source for humans to large doses of IR. Current estimates of lifetime cancer risk are around 40% and 50% for the USA and the UK, respectively [10–12]. Since RT is administered to roughly half of all cancer patients [13], a significant fraction of the population in most Western countries will be treated with IR, mostly with curative intent [14, 15]. This produces a large population susceptible to experience chronic radiation toxicity. And despite engineering efforts to deliver RT specifically to tumors, neighboring healthy tissue is also damaged, which in turn impacts patient morbidity and quality of life [16–18].

The side effects or ‘normal tissue damage’ manifest in two phases: an early (acute) radiation syndrome (‘radiation sickness’) that arises hours to days after treatment and can subside within weeks; and a late (chronic) radiation toxicity (LRT) which can persist for months to years after treatment completion [16, 19]. Acute effects depend on the specific radiation parameters (dose, dose rate and type of radiation) and the tumor location, but are ubiquitous among RT patients and include fatigue, bleeding, dryness, vomiting and diarrhea, fever, hair loss and inflammation [16]. This early radiation sickness reduces the quality of life of patients, but can be well managed in a modern healthcare system [16, 20]. However, the pathogenesis of LRT is poorly understood, making treatment or even prediction difficult. Like early side effects, late ones depend on irradiation site and absorbed dose, and can include cardiovascular disease, neuronal damage, chronic inflammation, fibrosis, tissue atrophy and secondary malignancies [16, 21–24]. Radiation-induced fibrosis, which can develop in the skin, subcutaneous tissue, lungs, intestine and urinary tract, is particularly difficult to manage, and is characterized by extensive extracellular matrix (ECM) deposition. This ECM remodeling eventually reduces the functional volume of the organ [25] and, in glandular/tubular organs, compromise a segment, which can lead to obstruction and functional collapse of the whole system [26]. Thus, radiation toxicity can severely reduce the quality of life of cancer patients and, in extreme cases, be fatal [27]. This burden on health care systems will only increase in the coming decades.

Currently, RT regimes are adjusted to keep the fraction of patients suffering from severe LRT at acceptable levels [26, 28, 29]. However, this means some patients will be prescribed suboptimal doses for tumor control. The ability to predict an individual’s radio-tolerance would increase the curative potential of RT for less sensitive patients and spare radiation-sensitive patients the chronic symptoms of late toxicity. However, for stratified/personalized RT to become reality, a better understanding of the biology underpinning radiation toxicity is required.

**Fig. 1:**
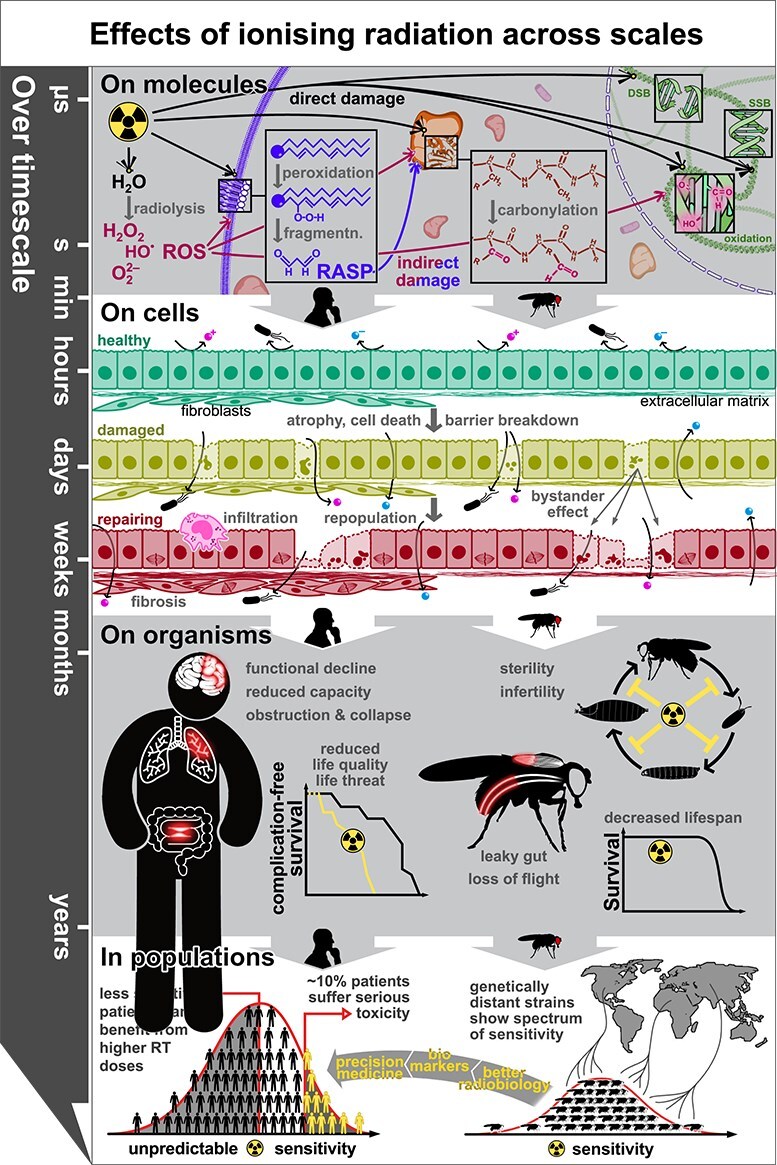
Effects of IR across time and biological scales in both humans and *Drosophila*. First row (on molecules): The first moments (from microseconds to a few seconds) the interactions of radiation with living matter are dominated by universal biochemical events: direct impact of radiation with water and macromolecules leads to ionization, oxidation and fragmentation. In proteins, this leads to carbonylation; in DNA, to damage of nitrogen bases or the sugar-phosphate backbone, both of which can result in single- or double-strand breaks (SSBs and DSBs, respectively). The radiolysis of water produces various ROS, while the oxidation of lipids leads very often to peroxidation and fragmentation that produces reactive aldehyde species (RASP). ROS and RASP further oxidize lipids, proteins and nucleic acids, possibly forming a positive feedback loop that could perpetuate the damage. All organisms have mechanisms to repair this damage that are not shown here. Second row (on cells): Schematic lateral view of a human (left, with fibroblasts and ECM) and fly (right, with ECM only) epithelium, over time. The healthy epithelium shows barrier function against molecules and bacteria, whereas an epithelium irradiated recently loses cells to apoptosis and has a compromised barrier; individual damaged cells influence the behavior of neighbors. Damaged tissue attempts repair through proliferation but further damage accumulates through bystander effects. At this stage, in humans, tissue inflammation and infiltration of immune cells may become chronic, and fibroblasts proliferate and produce additional ECM. Third row (on organisms): Tissue chronic fibrosis and inflammation lead to functional decay, which in humans can significantly reduce quality of life and, in some cases, be fatal. In flies, radiation reduces overall organismal fitness, negatively impacting developmental completion, fertility and lifespan, with some tissues showing specific dysfunction. At this scale, damage is irreversible. Fourth row (in populations): Human sensitivity to chronic radiation damage is a complex polygenic trait that cannot be predicted at the individual level; this is a major limitation to cancer RT. *Drosophila* radiosensitivity also seems to have a genetically complex basis, which means it could help better understand radiobiology and thus inform ‘precision medicine’ approaches in radiotherapy.

The irradiation of living cells and tissues initiates a sequence of events that spans biological scales, starting at the molecular and cellular level and extending up to the tissue and whole-organism level. We argue that the sequence of events induced by exposure is sufficiently conserved between *Drosophila* and humans for research in the fly to provide insights into normal tissue toxicity (Fig. 1, bottom row).

## *DROSOPHILA*’S POTENTIAL FOR RADIOBIOLOGY RESEARCH

**Fig. 2:**
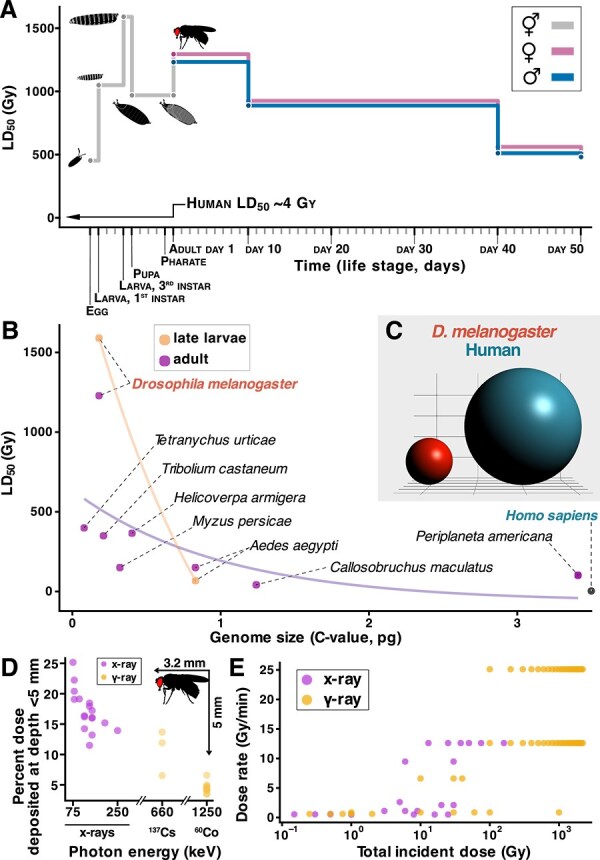
Radio-resistance in *Drosophila* is due at least partially to genome and body size. (A) The median lethal dose (LD_50_) of IR for *Drosophila* is strongly stage- and age-dependent, and is consistently 100–300 fold higher than for humans. *Drosophila* LD_50_ data of *Canton S* wild-type strain reared at 25°C [32, 36]. (B) Median lethal doses for various insect species plotted against their genome size in C-value. Genome size correlates with radiosensitivity. Lines are exponential curves fitted to the larval and adult data. LD_50_ values are from the International Database on Insect Disinfestation and Sterilization (IDIDAS) of the International Atomic Energy Agency; C-values are from the Animal Genome Size Database [37], or estimated from genome length. (C) Visual comparison of human and *Drosophila* nuclear volumes, assuming this is proportional to diploid genome length. (D) Dependence of the radiation energy deposited in the first 5 mm of target depth (expressed as % of the total deposited energy) on the energy of the incident photons. Values obtained from ‘percent dose depth’ (PDD) curves or data tables in [38–45]. Where data tables were not available, numerical values were extracted from plots using WebPlotDigitizer. These values were manually integrated for the first 5 mm to obtain the % of total dose deposited in that depth interval of the beam path. These integrated values are certainly overestimated, as all empirical and simulated PDD calculations are usually truncated before all energy has been deposited. (E) Range of incident doses and their dose rates in studies aimed at identifying genes involved in *Drosophila* radiosensitivity [32, 46–52], showing the disparity between assays. Further disparity arises from the nature of radiation; here we only highlight those doses administered as X-rays (typically ~100 to ~300 keV) or γ-rays (typically ~660 keV and over).

Insects are generally considered to be extremely radio-resistant, with adult *Drosophila* being ~300-fold more resistant than humans to γ-radiation under whole-body irradiation (WBI) conditions [30, 31] (Fig. 2A). This insect radio-resistance has been attributed to their life history (high fecundity and short lifespan); indeed, adult insect tissues are short-lived, mostly postmitotic and therefore spared from radiation-induced mitotic catastrophe [32–34]. Thus, *Drosophila* do not experience the hematopoietic nor gastrointestinal syndromes observed in humans who have undergone WBI [35]. However, this explanation of insect resistance is challenged by the observation that *Drosophila* larval stages, with high mitotic activity are more, not less, radio-resistant than adults (Fig. 2A).

We argue that the perceived radio-resistance of insects is largely due to both their reduced genome and small body sizes. A larger genome occupies a larger volume and so has a higher likelihood of collision with IR photons or particles and receive damage. In plants, it is well established that radiation-induced DNA damage is inversely proportional to genome size [53–55]. Comparing genome size and radiation sensitivity (LD_50_ survival) between insect species shows the same inverse correlation (Fig. 2B). With this in mind, it is worth considering that the *Drosophila* genome is ~36 times smaller than the human genome (diploid genome sizes of 175 Mb and 6.27 Gb, respectively) [56, 57]; if this difference were represented in perfectly spherical nuclear volumes, a *Drosophila* nucleus would have a ~2.5 times smaller radius than a human (Fig. 2C), making collisions with IR less likely.

Dosimetry could be another factor contributing to the perception of *Drosophila* as radio-resistant. Comparisons between species are established respect to the incident dose, which is straightforward to determine. However, biological damage better correlates with the absorbed dose, with the amount of damage received dependent on both the physical properties of the irradiated object and the incident dose of radiation [58, 59]. For instance, the greater the energy of incident photons, the deeper the path along which their energy is released and thus, the smaller the fraction of energy deposited at the surface of the target (Fig. 2D). Therefore, *Drosophila*, with a body thickness of ~1 mm, is likely to receive a greater absorbed dose from low-energy X-rays than from the same incident dose in the form of ^137^Cs or ^60^Co γ-rays [60].

Taking into account the life history, genome and body size, and limited dosimetry work in *Drosophila*, it is plausible that the maximum ‘absorbed’ doses tolerated by *Drosophila* are far closer to those of humans. In addition, *D. melanogaster* offers itself as an attractive pre-clinical *in vivo* system to study the cellular and molecular biology of the late radiation response. First, *Drosophila* has a long history in radiation research, beginning with H. J. Muller’s demonstration that X-rays induce mutations, which won him the Nobel Prize in 1946 [61, 62]. Further, *Drosophila* rearing is inexpensive and has a short lifespan (2–3 months), making it well-suited for research into late-onset pathologies using large cohorts. Most importantly, the tools for genetic manipulation in *Drosophila* are highly developed [63], and considering its genome harbors orthologues to ~75% of all known human disease-causing genes [64], this allows identification of new molecular mechanisms associated with LRT that may be relevant to human biology. *Drosophila* is ideal for unbiased genetic screens using reverse genetics approaches or genome-wide association studies (GWAS) using genetic reference panels [65]. By contrast, GWAS of patients are long, expensive, and have yet to provide full mechanistic insight of the radiation response [21, 66]. Finally, the biochemistry that underpins the radiation response (oxidative biology) and the subsequent cellular processes that are activated (DNA damage response and redox homeostasis) are highly conserved [67, 68]. These points highlight the capacity of *Drosophila* as a genetic model organism—but how similar is *Drosophila’s* radiation response to that of humans?

## BIOLOGICAL SCALES OF RADIATION DAMAGE

### Immediate molecular damage

Incident photons or particles interact with the target matter in various ways, ultimately resulting in electrons escaping their orbits, leaving behind charged atoms and molecules [69]. Where escaped electrons are part of a covalent bond, this will break, changing the properties of molecules and forming free radicals [70]. Multiple free radical species arise from the radiolysis of water (•OH, •H, •O_2_H, H_3_O^+^, OH^−^, H_2_O_2_, H_2_); these reactive oxygen species (ROS) oxidize and damage biomolecules [71, 72]. Oxidative damage also arises from the direct ionization and breakage of biomolecules, which form more free radicals. In linear polymers such as nucleic acids, proteins and many lipids, this results in polymer fragmentation and oxidation (Fig. 1, top row).

As the cell undergoes widespread oxidation, the stress induces a robust antioxidant response. This involves expression or activation of multiple cellular enzymes that reduce ROS directly (Catalase, Superoxide Dismutase) or through cycles of glutathione oxidation–reduction (Glutathione S-transferases) [73]. These enzymes are very efficient and can restore normal oxidation levels within hours of oxidative challenge. However, radiation-induced oxidative stress can persist, accumulating ROS throughout the lifetime of the cell. This has been shown both *in vitro* and *in vivo*, with *in vivo* studies indicating persistence for years [74–76]. In cancer patients, chronic oxidative stress is thought to contribute, alongside chronic inflammation, to LRT [77, 78], although the limits of human research have prevented proving this conclusively [79].

Though direct observations of radiation-induced ROS have yet to be reported in the fly, multiple studies have identified that exposure leads to sustained expression of genes involved in oxidative metabolism. Both *Glutathione S transferase T4* (*GstT4*) and *Glutathione S transferase D1* (*GstD1*) have been shown to be upregulated after radiation treatment (ART) in adult flies [80, 81]. Moreover, prophylactic treatment of *Drosophila* with the antioxidant epicatechin improves radiation survival outcome [46, 82]. This suggests that, like in humans, irradiation of *Drosophila* leads to ROS accumulation and a transcriptional response to elevate levels of antioxidant enzymes.

Of all radiation-induced macromolecular effects, DNA damage is the most relevant to radiobiology—though it has been proposed that protein oxidation provides a significant contribution [54]. DNA damage can modify individual nucleotides, with guanine being the nucleobase most sensitive to radiation; this type of damage results in either DNA adducts or single strand breaks (SSBs) [83]. Moreover, closely spaced ionizations can lead to double strand breaks (DSBs) and the fragmentation of chromosomes. Cells experience these lesions throughout their lives in the absence of radiation, so there are multiple biochemical pathways, conserved in flies and humans, that recognize and repair these lesions [67, 84]. Interestingly, while the molecular damage is almost instantaneous, and DNA repair usually takes a few hours, persistence of DSBs has been observed for several days after irradiation, both in flies and human cells [33, 85].

Further, the spatial distribution of DNA lesions along a strand has been shown to cluster in so-called ‘complex DNA damage’, possibly arising from the dense tracts that ionizing particles travel along through the cell nucleus. These clusters, which can be composed of a combination of SSBs, abasic sites and base damage (e.g. oxidized nucleotides), have the potential, if left unrepaired, to become DSBs. These DSBs can then either be correctly repaired through non-homologous end-joining or incorrectly repaired, creating cell-lethal mutations such as chromosomal rearrangements [86–88]. Though we suspect that IR would induce complex DNA damage in the fly, this has yet to be directly observed; this finding would support the fly as a preclinical model of radiation toxicity.

### Cellular and tissue dysfunction

Excessive and unrepaired DNA damage after irradiation can lead to dysfunction and cell death through multiple processes [75, 89], including apoptosis [90], mitotic catastrophe [91] or induced senescence [92]. The pathway taken by a cell will depend on its inherent repair and proliferative capacity, though most radiation-induced cell death seems associated with p53 activation [93]. This variability in the response is compounded by cell-to-cell heterogeneity in absorbed dose, and as a result, irradiation will only damage a proportion of the exposed cells, and of these, only a fraction will be beyond repair and die [94]. Cells uninjured or with sub-lethal damage will attempt tissue repair through cell proliferation, to recover cellularity, or stimulate fibrosis (Fig. 1, second row). However, damaged cells release signaling molecules that can instruct unharmed cells to undergo apoptosis (the ‘bystander effect’) and secrete in turn further signaling molecules, many with pro-inflammatory roles. Other cells may become senescent, unable to help regeneration, but chronically secreting signaling factors that affect other cells [95, 96]. While the cell-autonomous, ‘biochemical’ repair can take place within hours, these non-cell-autonomous responses occur over days or weeks, and their dependence on proliferation means that the effects of radiation vary strongly between target tissues and among their different cell types. In this period, additional complexity arises, as irradiation causes vascular damage, leading to reduction in oxygen supply, as well as inflammation and infiltration of immune cells [16, 97]. This inflammation can persist for months and years after cellularity is recovered, becoming chronic and leading to fibrosis [27]. But the perturbation of the signaling environment of the damaged tissue is not only the result of the DNA damage response. ROS also act as signaling molecules, so cells try to maintain ROS in the picomolar range to not saturate signal transduction pathways [68, 98, 99]. The deregulation of ROS levels interferes directly with ROS-mediated signaling and indirectly with other signaling pathways by altering the function of proteins and lipids through their oxidation. The effects of chronic oxidative stress on signaling pathways are still actively studied [100, 101]. This interplay of signaling, cell types and tissue architecture over months is very difficult to model either *in vitro* or *ex vivo* from patient-derived samples.

In the *Drosophila* larva, irradiation can lead to widespread apoptosis in the highly proliferative imaginal disks—the epithelial primordia of the adult epidermis [102]. This leads to a robust regenerative response that has been extensively studied, with the mechanisms connecting DNA damage, apoptosis and compensatory proliferation have been largely identified [103–105]. However, long-term effects have been reported, and little is known about the mechanisms behind them; irradiation of larvae leads to an increase in the number of adult brain neurons expressing the activated effector caspase *Dcp-1* (orthologue to human *CASP3/7*) [47]; irradiation of adult flies leads to both the adult fat body (adipocytes) and intestine expressing inflammation-like markers 2 weeks later (which is a long time for *Drosophila*), and this correlates with tissue dysfunction, such as loss of epithelial barrier [33]. As in humans, tissue responses vary: the adult gonads—the most proliferative *Drosophila* tissue in adulthood—are irreversibly obliterated upon irradiation [48]; within imaginal disks, local signaling determines the resistance to irradiation-induced apoptosis [106]. Additional evidence of tissue specificity is that genes associated to the *Drosophila* radiation response (Table 1) have tissue-specific expression, suggesting that their protective role is restricted to those tissues; moreover, radiation of adult *Drosophila* induces autophagy 1 day ART (likely to clear away oxidized material), but only in the midgut and the brain [107].

**Table 1:** List of known *Drosophila* genes that have been experimentally validated to be involved in the radiation response. For each gene the known biological function is provided as well as references for the radiobiological studies it was identified from.

Gene	Symbol	Main function	Identification approach	Reference	Gene ID (FlyBase/ENSEMBL)
*Drosomycin*	*Drs*	Antimicrobial response (effector)	GWAS, transcriptomics	[33, 62]	FBGN0283461
*Drosomycin-like 1*	*DroA*	Antimicrobial response (effector)	GWAS, transcriptomics	[33, 62]	FBGN0052274
*Diptericin A*	*Dipt*	Antimicrobial response (effector)	GWAS, transcriptomics	[33, 62]	FBGN0004240
*Attacin-C*	*AttC*	Antimicrobial response (effector)	Transcriptomics	[62]	FBGN0041579
*Cecropin C*	*CecC*	Antimicrobial response (effector)	Transcriptomics	[62]	FBGN0000279
*Metchnikowin*	*Mtk*	Antimicrobial response (effector)	Transcriptomics	[62]	FBGN0014865
*Death caspase-1*	*Dcp-1*	Apoptosis (effector)	Candidate	[90]	FBGN0010501
*reaper*	*rpr*	Apoptosis (regulator)	GWAS	[33]	FBGN0011706
*head involution defective*	*hid*	Apoptosis (regulator)	GWAS	[33]	FBGN0003997
*SMAD specific E3 ubiquitin protein ligase*	*Smurf*	BMP/TGFbeta signaling	GWAS (below GW significance)	[94]	FBGN0086299
*E2F transcription factor 1*	*e2f1*	Cell cycle (regulator)	Candidate	[120]	FBGN0011766
*Hus1-like*	*hus1-like*	Cell cycle checkpoint response (regulator)	Candidate	[106]	FBGN0026417
*DNA ligase 4*	*DNAlig4*	DNA repair (effector)	Candidate	[98]	FBGN0030506
*rad54*	*okr*	DNA repair (effector)	Candidate	[98, 106]	FBGN0002989
*meiotic 9*	*mei-9*	DNA repair (effector)	Candidate	[63, 106]	FBGN0002707
*Recombination repair protein 1*	*Rrp1*	DNA repair (effector)	Candidate	[106]	FBGN0004584
*Ku80*	*Ku80*	DNA repair (effector)	Candidate	[106]	FBGN0041627
*WRN exonuclease*	*WRNexo*	DNA repair (effector)	Candidate	[106]	FBGN0038608
*Bloom syndrome helicase*	*Blm*	DNA repair (effector)	Candidate	[106]	FBGN0002906
*Xeroderma pigmentosum, complementation group C*	*Xpc*	DNA repair (regulator)	Candidate	[63, 97]	FBGN0004698
*mutagen-sensitive 302*	*mus302*	DNA repair (regulator)	Candidate	[131]	FBGN0287696
*BRCA2*	*Brca2*	DNA repair (regulator)	Candidate	[106]	FBGN0050169
*spindle B*	*spn-B*	DNA repair in meiosis and mitosis (effector)	Candidate	[65, 106]	FBGN0003480
*loki*	*lok*	DNA repair, apoptosis (regulator)	Candidate	[106]	FBGN0019686
*p53*	*p53*	DNA repair, Apoptosis, Cell cycle checkpoint response (regulator)	Candidate	[120]	FBGN0039044
*meiotic 41*	*mei-41*	DNA repair, Cell cycle checkpoint response (regulator)	Candidate	[106]	FBGN0004367
*grapes*	*grp*	DNA repair, Cell cycle checkpoint response (regulator)	Candidate	[108]	FBGN0261278
*Proliferating cell nuclear antigen*	*PCNA*	DNA repair, replication (regulator)	Candidate	[106]	FBGN0005655
*spineless*	*ss*	Gene expression - Cell fate specification (regulator)	Candidate	[63]	FBGN0003513
*pannier*	*pnr*	Gene expression - Cell fate specification (regulator)	GWAS (below GW significance)	[94]	FBGN0003117
*musashi*	*msi*	Gene expression - Cell fate specification, Normoxia (regulator)	GWAS	[33]	FBGN0011666
*NK7.1*	*NK7.1*	Gene expression (regulator)	GWAS (below GW significance)	[94]	FBGN0024321
*Discoidin domain receptor*	*Ddr*	Neural axon formation	GWAS (below GW significance)	[94]	FBGN0053531
*Superoxide dismutase 1*	*Sod1*	Redox homeostasis	Candidate	[65]	FBGN0003462
*Cytochrome P450 6 g1*	*Cyp6g1*	Redox homeostasis	Candidate	[65]	FBGN0025454
*Glutathione S transferase T4*	*GstT4*	Redox homeostasis	Candidate	[65]	FBGN0030484
*Glutathione S transferase D1*	*GstD1*	Redox homeostasis	Transcriptomics	[62]	FBGN0001149
*Connector of kinase to AP-1*	*Cka*	Signaling	GWAS	[33]	FBGN0044323
*Growth arrest and DNA damage-inducible 45*	*Gadd45*	Stress and DNA damage response (signaling)	Transcriptomics, Candidate	[62, 65, 106]	FBGN0033153
*Heat-shock-protein-70*	*Hsp70*	Stress response (effector)	Transcriptomics, Candidate	[62, 65]	FBGN0286924
*lincRNA.1043*	*CG14621*	Unknown	GWAS (below GW significance)	[94]	FBGN0031183
*CG42324*	*CG42324*	Unknown	GWAS	[33]	FBGN0259224
*CG1824*	*CG1824*	Unknown	GWAS	[33]	FBGN0030403

### Organismal effects of radiation

In flies and humans, exposure to IR reduces lifespan in a dose-dependent manner. In both organisms, radiosensitivity depends on demographic variables such as age and sex, with males and older individuals being more sensitive [30, 32, 33, 46, 81, 109]. In adult *Drosophila,* where most tissues are postmitotic, the age-dependency of radiation sensitivity strongly suggests that mechanisms other than mitotic catastrophe influence survival. While there has been rather little exploration into the molecular and cellular bases of lifespan shortening after irradiation, it seems that DNA repair genes are essential for radiation tolerance, as mutations in DNA repair genes such as *mei-9* (orthologue to human *ERCC4*; excision repair), *mei-41* (orthologue to *ATR*; broad DNA repair and cell cycle signaling), *DNAlig4* (orthologue to *LIG4*; non-homologous end joining) and *okra* (orthologue to *RAD54L*; homologous recombination) negatively affect survival after radiation exposure [49, 50, 110, 111].

Currently, the most widely accepted model to describe how radiation affects biological systems is the ‘linear, no-threshold’ model, whereby ‘any’ exposure is damaging and the strength of biological effects increases with dose. However, this model does not explain radiobiological and epidemiological studies that describe positive effects of low IR exposure [112–114]. Many studies (mostly in human cells *in vitro*, but also with rodents) have suggested a range of metabolic, immunological and regulatory mechanisms whereby low-dose IR induces an adaptive response that can protect against higher dose radiation and possibly other, generic insults [75, 115–117]; but how these mechanisms apply long-term *in vivo* is less clear and technically difficult to address. The existence of a biphasic response (hormesis) in humans would have far-reaching implications in radioprotection, occupational health and medical practice, but definitive proof would require extremely large epidemiological cohorts [118] and therefore remains controversial. *Drosophila* seems to have a biphasic dose–response whose underlying mechanisms have been explored to some extent. For instance, irradiation of oocytes with 0.02 Gy makes them more resistant to a subsequent exposure to 2 Gy [111]. This desensitizing effect was dependent on the DNA repair genes *mus302* (orthologue to *RFWD3*) and *mei-41* (DNA repair and cell cycle signaling). Adult lifespan seems to have a similar hormetic dose–response [119]; exposure to γ-radiation throughout development (0.4 Gy accumulated dose) had radio-protective effects against a 30 Gy treatment in adulthood. This protection was also dependent on DNA repair genes (*mus209*, *mus210*, *spnB* and *okra*). While the conventional explanation for these effects is the establishment of some transcriptional memory that keeps protective genes expressed, this has not been proven yet. Transcriptomics studies on *Drosophila* have attempted to correlate low radiation dosages (within a 0.1–200 Gy range) with persistent gene expression changes, but have found only transient changes [120]. Nevertheless, it is clear that *Drosophila* can make relevant, mechanistic contributions to understanding the dose–response to IR.

Besides impacting mortality risks, IR negatively affects organismal biology through neurological effects. Prenatal survivors of the Hiroshima and Nagasaki atomic bombs had a higher prevalence of mental retardations and lower performance on reasoning tests at school [121]. Likewise, large cohort studies have revealed that irradiation of the brain during early infancy can lead to deficits of learning and logical reasoning in adulthood [122]. Experimental mouse models show that radiation reduces neurogenesis in the adult hippocampus, accompanied by widespread apoptosis; this was associated with memory defects [123]. Similarly, in *Drosophila*, irradiation negatively influences memory formation in adults [80], and larval exposure to IR leads to sustained levels of apoptotic neurons in the adult brain [47]. This was accompanied by increased levels of antimicrobial peptides in the adult brain, suggesting chronic activation of innate immunity ART. Lastly, in irradiated larvae, neuronal neurotransmitter sensitivity was found to be dysregulated [124]. This indicates that radiation toxicity not only kills neurons but also leads to neuronal misfiring; both likely contribute to the observed cognitive decline. Central nervous system sensitivity to radiation seems to be conserved from mammals to flies, and fly research is beginning to point to a possible mechanism involving conserved pathways of cell death and inflammation.

## RADIATION DAMAGE IN *DROSOPHILA*

### Radiation toxicity is specific to life cycle stage

*Drosophila* starts life as an egg, which hatches into a larva that will eat and grow from ~1 μg to ~1 mg in 5 days, with extensive proliferation. Then it will form a pupa that undergoes metamorphosis for about 5 more days, with extensive tissue remodeling and destruction and takeover. After that, an adult (imago) emerges with its definitive size, a mostly post-mitotic soma, and the aim to reproduce. *Drosophila* developmental stage appears to be the greatest determinant of radiation treatment severity (Fig. 2A) [32]. Irradiation during the early larval stages leads to extensive cellular loss and regeneration, which imposes a developmental delay [125] (Fig. 2A). The irradiation of adults clearly induces harmful effects: the adult midgut shows widespread DSBs that from 30 minutes to several days ART while the animals displayed reduced locomotor activity one day ART [33]; another study also observed loss of flight capacity one day ART [109].

Though *Drosophila* has not been used formally to model chronic radiation toxicity, it exhibits some late radiation effects, like the loss of intestinal barrier function up to 14 days ART (15–20% of *Drosophila* adult lifespan). The most compelling late effect, however, is the shortening of lifespan [32, 33, 80, 110], which interestingly, occurs regardless of the developmental stage of irradiation [47]. This suggests that neither the regeneration in larval stages nor tissue remodelling during metamorphosis can repair the radiation damage. This indicates that irradiated adult *Drosophila*, with tissues of limited regenerative capacity, suffer sustained radiation damage and toxicity. This further points at the similarities between radiation toxicity in humans and flies, both in the processes that dominate the short-term response (DNA damage and apoptosis) and those that lead to tissue-level dysfunction (locomotion, epithelial barrier and intestinal atrophy) (Fig. 1).

### The genetic architecture of LRT: the view from *Drosophila* so far

The potential benefits of predicting the radiosensitivity of cancer patients have been discussed above and in more detail elsewhere [16, 26, 29]. Some efforts have focused on developing *ex vivo* diagnostic tests using patient-derived cells to predict patient radiosensitivity ahead of treatment planning [126, 127]. However, the tissue-specificity of radiosensitivity and the complexity of the LRT limit the accuracy of this approach [128–130]. Considering that the human radiation response is believed to be highly polygenic, the radiogenomics community has focused on finding genomic biomarkers for LRT [21, 29]. Multiple GWAS have been performed to identify genetic variants associated with LRT in the hopes of obtaining a panel of polymorphisms that will allow the stratification of patients [21, 131]. After ~15 years of research, the first clinical trials for this approach to predict late complications of radiotherapy of breast cancer are starting [130].

The history of *Drosophila* as a research organism has uniquely equipped it for the identification of genes underpinning a trait. While *Drosophila* radiosensitivity has not been explored to saturation yet, multiple studies have cumulatively identified at least 43 genes involved in the radiation response (Table 1) and suggest that the radiation response of *Drosophila*, like in humans, is a genetically complex trait. These studies also highlight the range of approaches available to *Drosophila* research:

#### Candidate approach

Twenty-two genes in the list were investigated because of their previously known functions. The influence of excision-repair pathway member *mei-9* (orthologue of *ERCC4*) on survival and induction of DNA damage ART has been studied from embryonic to adult stages. *Mei-9* is the most validated gene in Table 1 involved in protection against IR damage [48, 49, 110, 119].

#### Genome-wide association

Two GWASs have been performed and only one of these studies identified any (five) significant genes [33, 109], both using the *Drosophila* Genetic Reference Panel (48). One measured flight ability 1 day ART, but failed to find any associations at genome-wide significance [109]. The authors concluded that the genetic panel was underpowered; however, the assay appeared saturated, with half the strains displaying 100% flight loss, likely due to the high dose given (1382 Gy). The other study used the loss of intestinal barrier function as a measure of radiation toxicity, finding six significantly associated polymorphisms in five genes [33]. One of these genes, *musashi* (*msi*, orthologue to human *MSI2/1*), was functionally validated and shown to regulate intestinal stem cell function. The mouse orthologue *Msi-1* has been shown to be involved in gut regeneration ART [132], showing the conservation of the radiation response between insects and mammals.

#### Transcriptomics

Two studies characterized the genome-wide transcriptional response of *Drosophila* ART [81, 120]. The first study described the effect of X-rays (1–200 Gy) at various timepoints (2–20 days ART) using whole-body mRNA sequencing [120]. Six genes were found differentially expressed at 20 days, of which *Connector of kinase to AP-1* (*Cka*) was previously identified in a fly GWAS [33]. While *Cka* was not successfully validated in functional tests in midgut enterocytes, its identification with orthogonal methods suggests that *Cka* might function in another cell type or tissue. The other transcriptomic study focused on the early response (1 day ART) to 144–864 Gy doses, aiming to observe dose-dependent transcriptional changes [81]. Both studies found very few differentially expressed genes, but independently identified *Growth arrest and DNA damage-inducible 45* (*Gadd45*, orthologue to human *GADD45G/A/B*). *Gadd45* is involved in the DNA damage response and has been functionally validated for its radio-protective role in *Drosophila* [119]. Lastly, a third work focused on the expression of small non-coding RNAs (ncRNAs) regulated by E2F and p53 after X-ray irradiation of larvae [133]. They found that *miRs 318, 932, 956, 966, 968–1002, 986* and *990* are regulated as part of the DNA damage response, and their individual loss of function leads to larval radio-sensitivity. Interestingly, it was also found that E2F and p53 regulate different groups of ncRNAs after DNA damage to larvae.

These studies demonstrate that the *Drosophila* radiation response (i) is a complex genetic trait, (ii) is evolutionarily conserved and (iii) has distinct short- and long-term transcriptional responses. It is intriguing that there is no overlap between the genes identified through orthogonal approaches i.e. none of the genes identified by the candidate approach were found differentially expressed in the transcriptomic studies. This likely reflects the complexity of the genetic architecture of radiosensitivity. Similar lack of overlap is found in radiogenomics studies with patients [134], again suggesting that we have just scratched the surface of the molecular and cellular elements that underpin organismal radiosensitivity.

## TOWARD A *DROSOPHILA* MODEL OF RADIATION TOXICITY

Current pre-clinical models of radiation toxicity that can address the complexity of late effects are mostly based on mice, rats and zebrafish, and have been reviewed elsewhere [135–141]. These models have the advantage of having a similar histological organization of tissues to humans and a higher degree of genetic conservation with us. Where small-animal radiotherapy instruments are available, rodents also allow mimicking the conditions in which radiation damage is most often delivered to humans. However, they also share some of the difficulties: protracted end-points (with their added expense) and difficulty to work with large number of individuals. While *Drosophila* does not have these drawbacks, it does have its own set of challenges.

### Histological and immunological differences

In humans, irradiation leads to a persistent immunological response that leads to two significant complications: fibrosis and chronic inflammation. To model these in *Drosophila*, whose immune and connective systems differ substantially from their human counterparts, some considerations are necessary.

#### Inflammation

In humans, radiation-induced ROS and oxidized macromolecules can recruit and activate immune cells [142]. Activated neutrophils can, in turn, produce and release H_2_O_2_ as part of their normal immunological activity [143]. It is easy to envision a positive feedback loop of ROS driving chronic inflammation and toxicity in RT patients, which is supported by the fact that neutrophils are particularly radiosensitive [144]. *Drosophila* hemocytes, similarly to neutrophils, are recruited to wounds via conserved damage-associated molecular patterns [145] which include extracellular H_2_O_2_ generated by damaged epithelial cells [146]. It is conceivable to think that IR may induce a similar epithelial response, leading to hemocyte recruitment. While in *Drosophila* there are no clear orthologues to the mammalian cytokines and chemokines that orchestrate radiation-induced inflammation, and the immune response does not induce swelling, its humoral immunity is remarkably conserved with that of humans [147, 148]. Moreover, several immune genes influence *Drosophila* radiosensitivity (e.g. *Drs*, *DroA*, *AttC*, see Table 1), suggesting that the fly could be used to investigate the molecular circuitry that connects radiation to the inflammatory response.

#### Fibrosis

In humans, irradiated tissues recruit monocytes, which differentiate into macrophages that later recruit fibroblasts [149, 150]. Activated fibroblasts proliferate and deposit excess ECM near the irradiated tissue, leading to fibrotic accumulation and strictures that can cause tissue dysfunction [151]. *Drosophila* lacks fibroblasts and mesenchymal tissue and therefore cannot develop ‘cellular’ fibrosis [145]. However, it can develop pathological fibrotic deposition of ECM components in the heart, nephrocytes, adipocytes and salivary glands [152–157], and therefore, it may be able to model this aspect of radiation toxicity, too.

### *Drosophila* body size imposes a WBI approach

Radiation toxicity in humans arises most often through radiotherapy delivered with an external beam [158], where the dose is targeted specifically to the tumor with a spatial precision of ~1 mm at most [159]. Most research irradiators are far from this sophistication and rely on WBI, which in vertebrate models can lead to lethal or very debilitating hematopoietic and gastrointestinal syndromes that prevent studies of late toxicity. This can be avoided to some extent using specialized shielding that only exposes the target organ, or altogether using expensive small-animal radiotherapy research platforms that have been developed in the last ~20 years [159, 160]. *Drosophila*, with a length of ~2 mm and thickness of ~1 mm, seems suitable only for WBI. This precludes entirely the study of rare systemic (abscopal) effects [161] of irradiation and of organ damage in isolation; on the other hand, *Drosophila* does not suffer catastrophic hematopoietic nor gastrointestinal syndromes, which makes still possible to study long-term, tissue-specific damage in the fly using WBI.

### Lack of reference dosimetry

A fundamental aspect of radiation toxicity is its dependence on ‘absorbed’ dose, dose rate and nature and energy of incident radiation. The observations reviewed here feature wide ranges of irradiation parameters, with dose-rate varying between 0.4 and 25 Gy/min, ‘incident’ dose between 0.5 and 1385 Gy (Fig. 2D–E) and photon energy between ~75 and 1250 keV. While the dependence of *Drosophila* survival on incident dose is clear, *Drosophila* radiobiological research lacks dosimetry studies to establish absorbed doses, and studies have rarely been performed with instruments that allow systematic exploration of the effects of dose rate or photon energy on the fly. Comparison across studies is difficult, as apart from differences in physical radiation parameters, lifespan is strongly affected by diet and genotype [162]. Reference dosimetry work would bring additional coherence and relevance to *Drosophila* radiobiology, and clarify whether *Drosophila* (and insects in general) truly have exceptional radioresistance. Proper dosimetry is a matter of increasing importance in radiation research, which has been highlighted in the context of environmental protection [163] and radiation biology with rodents [164].

### Advantages and outstanding questions

*Drosophila* allows outstanding experimental tractability, having the longest history of gene discovery [165, 166] and the capacity to seamlessly integrate genetic variation with molecular and cellular biology and physiology, in an organism with minimal footprint, short life cycle and adult lifespan, high fecundity and cheap rearing costs [167]. The parallels between the radiation responses of humans and *Drosophila* place it in an ideal position to address some of the outstanding questions in radiobiology. What sustains the development of late effects before they arise? Is it chronic oxidative stress, inflammation and DNA damage? How do they become chronic, if normal repair mechanisms can restore their molecular damage in hours? Does radiation really induce hormesis in the fly, and if so, what are the underlying mechanisms? These questions are relevant for human health and of intrinsic biological interest.
